# Middle Meningeal Artery Embolization as an Adjuvant Treatment for Bilateral Chronic Subdural Hematomas From Spontaneous Intracranial Hypotension: A Case Report

**DOI:** 10.7759/cureus.20609

**Published:** 2021-12-22

**Authors:** Guilherme Barros, Robert H Bonow, Michael R Levitt

**Affiliations:** 1 Department of Neurological Surgery, University of Washington, Seattle, USA

**Keywords:** case report, subdural hematoma, intracranial hypotension, middle meningeal artery, embolization

## Abstract

Middle meningeal artery embolization (MMAE) is an effective initial or adjuvant treatment for chronic subdural hematomas. However, its efficacy in the setting of spontaneous intracranial hypotension has not yet been reported. We present a case of progressive bilateral chronic subdural hematomas secondary to spontaneous intracranial hypotension that failed the initial lumbar epidural blood patch. The patient underwent surgical hematoma evacuation, with adjuvant MMAE and additional epidural blood patch, with good clinical and radiographic results. This case demonstrates the potential value of MMAE for chronic subdural hematomas secondary to spontaneous intracranial hypotension.

## Introduction

Chronic subdural hematomas (cSDH) secondary to spontaneous intracranial hypotension (SIH) can be challenging to manage. They are present in approximately 50% of SIH patients [1]. Despite epidural blood patches (EBP) to treat the underlying SIH and/or surgical cSDH evacuation, they have a significant rate of recurrence or progression [2-4]. Middle meningeal artery embolization (MMAE) has emerged as a standalone therapy or adjunct to surgical evacuation for cSDH. However, MMAE has not been previously reported as a treatment for cSDH specifically due to SIH. We describe a patient presenting with SIH and progressive bilateral cSDH, ultimately treated successfully with a combination of surgical evacuation, MMAE, and EBP.

## Case presentation

An otherwise healthy male in his 50s presented with several weeks of 8/10 intensity postural headaches. There was no history of trauma, spine surgery, lumbar puncture, pharmacologic anticoagulation, connective tissue disorder, or hereditary/acquired coagulopathy. Computed tomography (CT) of the head demonstrated bilateral cSDH, which mildly enlarged on follow-up.

**Figure 1:**
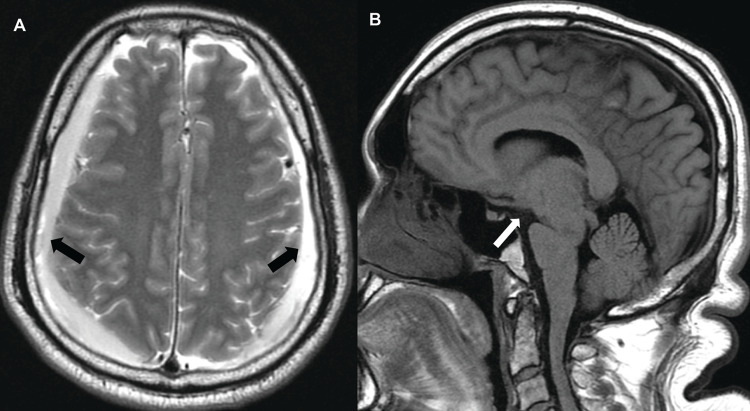
Initial consultation, non-contrast magnetic resonance imaging of the brain. (A) T2 axial plane demonstrating bilateral subdural hematomas (black arrows). (B) T1 sagittal plane demonstrating flattened pontomesencephalic angle (white arrow).

Following the initial CT, the patient underwent magnetic resonance imaging (MRI) of the brain and cervical, thoracic, and lumbar spine, including MRI myelography. MRI of the brain (Figure 1) redemonstrated bilateral cSDH, in addition to flattening of the pontomesencephalic angle.

**Figure 2:**
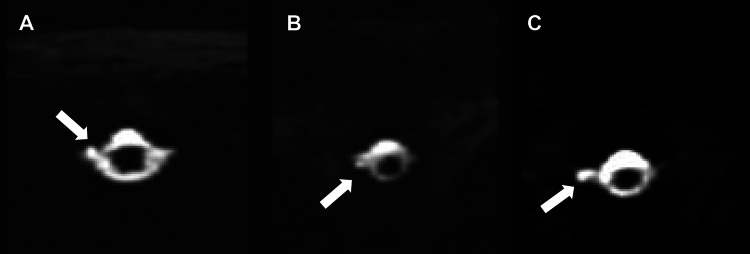
Axial reformatted MRI thoracic spine myelogram. Arrows show right-sided perineural cysts within the neuroforamina of (A) T4-T5, (B) T7-T8, and (C) T9-T10.

The MRI myelogram showed several right-sided thoracic neuroforaminal cysts, shown in Figure 2, with no definitive site of cerebrospinal fluid leak. Given the patient’s postural headaches, lack of history of head trauma, and imaging findings, there was high clinical suspicion for SIH.

**Figure 3:**
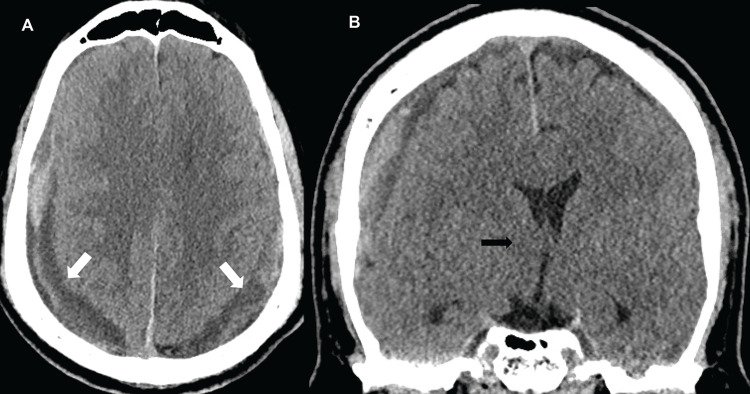
Emergency department presentation, non-contrast computed tomography of the head. (A) Axial plane. (B) Coronal plane. Enlarging bilateral subdural hematomas (white arrows) and right-to-left midline shift (black arrow).

The patient underwent a lumbar EBP as the initial treatment. This resulted in subjective improvement in headaches. A follow-up CT one week after the EBP showed mildly reduced cSDH size. Three weeks after EBP, CT showed interval growth of the cSDH. The patient presented to the emergency room several days later with new-onset transient perioral numbness and speech arrest, concerning seizure. Additional CT (Figure 3) showed right-to-left midline shift and further progression of the bilateral cSDH.

**Figure 4:**
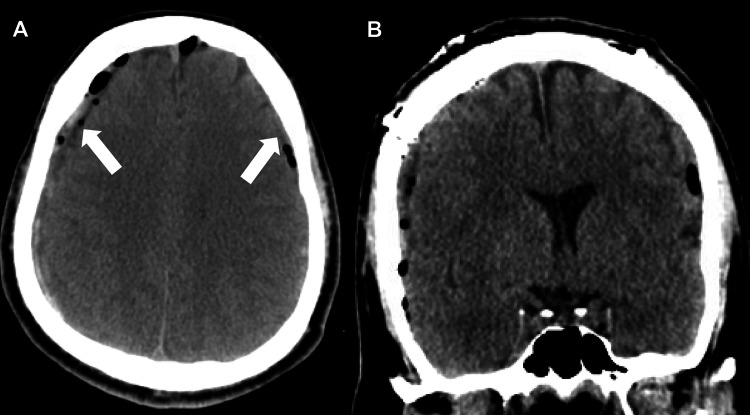
Immediate postoperative non-contrast CT of the head after right-side craniotomy and left-side burr holes for bilateral subdural hematoma evacuation. (A) Axial section and (B) coronal section showing small residual subdural hematoma bilaterally (arrows).

Levetiracetam monotherapy was initiated. The patient underwent simultaneous right-sided craniotomy and left-sided burr holes for cSDH evacuation. A small craniotomy was required on the right side after the initial attempt with burr holes was unsuccessful in evacuating the hematoma due to extensive membranes. Postoperative CT showed small residual cSDH bilaterally, shown in Figure 4.

**Figure 5:**
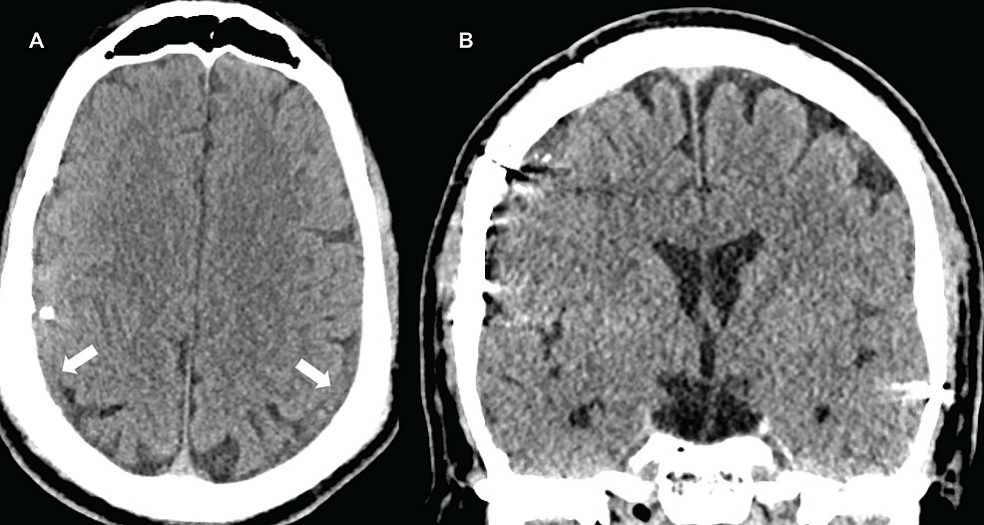
Six-week postoperative follow-up, non-contrast computed tomography of the head. (A) Axial plane. (B) Coronal plane. Minimal residual bilateral subdural hematomas (arrows) following surgical evacuation and middle meningeal artery embolization.

The next day, the patient underwent bilateral MMAE with Onyx, and on postoperative day two, he underwent a second lumbar EBP. These additional procedures were done following surgery to attempt a multimodal approach at treating his refractory subdural hematomas secondary to intracranial hypotension. The patient was discharged home on postoperative day three, with minimal headache and no further seizures. At six-week follow-up, the patient reported no headache and occasional seizure auras, with no actual seizures confirmed on an outpatient electroencephalogram (EEG). Head CT (Figure 5) showed significantly decreased bilateral cSDH with minimal residual volume.

## Discussion

Management of cSDH due to SIH often requires a multimodal approach. Initial conservative measures include bed rest, hydration, caffeine, and close follow-up brain imaging to ensure there is no progression [1]. However, this has limited clinical efficacy, with reports of only 18%-23% experiencing favorable outcomes [2,3]. Patients may undergo myelography to localize a potential source of cerebrospinal fluid leak, followed by first-line intervention with a blind or targeted EBP [4]. The effectiveness of single or multiple EBP alone for the treatment of cSDH due to SIH without surgery ranges from 63% to 72% [2-4]. Surgical evacuation of the cSDH is usually reserved for progressive enlargement, neurologic deterioration, and/or lack of improvement of SIH symptoms despite first-line therapy, as in the current case. In cases where surgical evacuation is performed as the initial treatment for the cSDH for SIH, the recurrence rate is especially high, with up to two-thirds of patients requiring additional evacuations with or without perioperative EBP [2]. However, surgical evacuation success is significantly improved if EBP is completed perioperatively, with up to 77%-85% exhibiting no cSDH recurrence and symptomatic improvement of SIH [3,4].

Further treatment modalities are needed to treat difficult-to-treat progressive or recurrent cSDH in the SIH setting, especially for those patients failing traditional first-line treatments. The use of MMAE for cSDH has not been reported specifically in the setting of SIH. Among a generalized cSDH patient cohort containing surgically naïve, recurrent after prior surgery, and adjuvant immediately following surgery, Link et al. reported 91.1% stable or decreased hematoma size without the need for further surgery after MMAE [5]. In a multi-center, prospective study with 138 patients undergoing MMAE as initial therapy or for recurrence after surgical evacuation, there was a 70.8% radiographic improvement of at least 50% volume reduction, with only 6.5% of patients requiring further cSDH treatments [6]. Comparing surgical evacuation of cSDH alone to surgical evacuation with adjuvant MMAE, MMAE improved mean volume reduction of the hematoma by 17.5 mL [7].

## Conclusions

In our patient with neurologic deterioration and radiographic progression of cSDH despite initial EBP, a multimodal treatment approach was employed. Surgical evacuation of the hematomas was indicated, and adjuvant MMAE with additional EBP likely assisted in preventing recurrence of the cSDH. Furthermore, the patient achieved immediate symptomatic improvement of the chronic headache, which was sustained at follow-up. This report highlights the novel use of adjunct MMAE for treating cSDH in the setting of SIH.
